# Promotion of Resilience in Migrants: A Systematic Review of Study and Psychosocial Intervention

**DOI:** 10.1007/s10903-021-01247-y

**Published:** 2021-07-29

**Authors:** Maria Ciaramella, Nadia Monacelli, Livia Concetta Eugenia Cocimano

**Affiliations:** 1 Department of Letters, Arts, History and Society, University of Study of Parma, Via Kennedy, 6, 43125 Parma, PR Italy; 2 Department of Economics and Business Sciences, University of Study of Parma, Parma, Italy

**Keywords:** Resilience, Migration, Intervention, Asylum seeker, Refugees

## Abstract

This systematic review aimed to contribute to a better and more focused understanding of the link between the concept of resilience and psychosocial interventions in the migrant population. The research questions concerned the type of population involved, definition of resilience, methodological choices and which intervention programmes were targeted at migrants. In the 90 articles included, an heterogeneity in defining resilience or not well specified definition resulted. Different migratory experiences were not adequately considered in the selection of participants. Few resilience interventions on migrants were resulted. A lack of procedure’s descriptions that keep in account specific migrants’ life-experiences and efficacy’s measures were highlighted.

## Introduction

In the past decades, migration has been dominating media and political discourses in Europe [1], as well as in the USA and Canada [2]. When discussing migrants and the impact of migration, we often refer to migrants as one group. This is partially due to the fact that migration policy creates a clear legal and then psychosocial boundary between the rights and freedoms of migrants and the population permanently residing in a country [3, 4]. For instance, from a legal perspective, an economic migrant is defined as person who leaves his/her country of origin purely for economic reasons in order to seek material improvements in his/her livelihood (European commission: Migration and Home Affairs). In the same perspective, a migrant becomes a refugee when he or she is recognised as a person who is forced to move and seek international protection. The person is recognised as being subject to a migratory movement in which there is an element of coercion, including threats to life and livelihood who requires protection from the host country. (United Nations Convention relating to the Status of Refugees adopted in Geneva on 28 July 1951).

Therefore, the legal criterion can categorise people regardless of their personal experience. Anyhow, like other groups within the population, migrants do not constitute a singular type with similar impacts, nor are migrant populations evenly distributed (or evenly received) across and within countries. These migrants come for a variety of reasons such as work, study, join or form a new family, receive protection from violence and persecution.

It can reasonably expect that all these different existential conditions involve very different experiences. Moreover, the history of migration illustrates the profound and multiple associations between migration and human vulnerability.

Although psychosocial suffering can characterise most of the migratory experiences, both individual or collective, the depth, pervasiveness and persistence over time of this suffering are linked to the specificity of each individual migratory experience. In particular, in our contemporaneity, the stressors that cause displacement are compounded by the risks of the migratory journey and the precarious living conditions in transit and arrival countries: uncertainty linked to asylum procedure and legal status, difficulties in accessing services, lack of work and economic difficulties, adequate housing, low resources to support oneself and one's family and social isolation. [5–7]. These factors, together with the loss of the original social environment and cultural meaning system, can contribute to the migrant's internal fragility (affecting the sphere of his or her thoughts, memory, affects, behaviours and future perspective) and external fragility (in his or her relations with others and the world).

Therefore, host countries find themselves having to face these important risks of fragility of the migrant population. Both the way in which migrants manage the integration process and the benefit they will bring to a given community depend on the severity of these risks and whether they can be solved. In this perspective, the type and quality of psychosocial interventions assume a relevant value for the entire community.

The construct of resilience, also understood in a broad sense as a key ability to thrive in the face of adverse and painful experiences that enables one to achieve a well-balanced state of psychosocial health [8], is undoubtedly a useful construct. In the variety of their approaches, resilience studies, on the one hand, provide numerous tools for studying the fragility/resources of migrant populations and, on the other hand, provide insights into intervention strategies to promote their psychosocial well-being and integration.

### Resilience and Migration

Indeed, resilience has aroused considerable interest in the migratory experience and several literature reviews proposed to systematise the results on the resilience process in the potentially multi-traumatic contexts of migration [9–12].

### Resilience and Psychosocial Interventions

In recent years, the WHO has endorsed the effectiveness of a range of scalable psychological interventions in particular for people suffering from mental health disease as disabling stress, depression and anxiety. A general psychosocial health, according to the WHO, could be obtained through interventions with a focus on the interrelation between individual life-history, i.e. people’s thoughts, emotions and behaviours, and social circumstances.

In their review, authors [13] analysed design intervention aimed at promoting resilience in different application contexts, taking into account the different definition of the construct of resilience proposed in the literature. The authors concluded that, regardless to the context, interventions based on an outcome-oriented definition of resilience are the most effective. Assuming resilience as a result of the interaction of protective factors, stressors and mental health status, ensures greater effectiveness of the intervention. This approach allows a pre-post evaluation of the dimensions involved in the resilience process and thus enables an assessment of the effectiveness of the intervention itself.

### Psychosocial intervention and migration

The literature on this issue is quite extensive. In order to account for the main challenges involved, we refer to two studies proposed at different times.

These two works [14, 15] highlighted critical gaps in research on the adequacy of clinical practice or psychosocial intervention programmes targeting the migrant population. In particular, the first of these works complained about insufficient attention to the cross cultural factors that should be included since the first ideation phase of psychosocial intervention. According to the authors, cultural background is an important factor that can influence the outcome of the intervention as a whole as well as that of the specific treatment. Individuals experience and manifest psychosocial disease or mental illness’s symptoms in different ways and diagnosis can change depending on cultures. Therefore, every aspect of the intervention, from planning to methodology design, from analysis to results’ discussion, have to be considered in light of changing cultural contexts. Thus, the development and testing of culturally sensitive psychosocial interventions in the migrant population is critical to the field.

More recently, a meta-analysis [15] revealed that psychosocial interventions had a clinically significant effect on PTSD, depression and anxiety outcomes. The authors underline the importance of this beneficial effect for populations exposed to continuous post-migration stressors. They concluded that, considering the epidemiological relevance of psychological distress and mental health conditions in refugees and asylum seekers, and in view of existing data on the effectiveness of psychosocial interventions, these interventions should be made routinely available as part of the health care of refugees and asylum seekers in distress.

## Aim

This review aimed to systematise papers reporting experiences of specific psychosocial interventions focused on promoting resilience among migrants in the host country.

The main questions that guided our analysis are the following:

1. The first question concerns the type of population that has been involved in the research. Migration experience, as mentioned above, can imply drastically different individual and collective experiences. Which of these different experiences are considered in the research, and why did the researchers involved this type of population?
2. The second issue concerns the theoretical framework and the consequent dimensions analysed by the researchers. The construct of resilience is far from a single definition and, as evidenced by a large body of literature, can be defined on the basis of very different theoretical paradigms. Different aspects have been studied, from the precursors of resilience, understood as personality traits, learned skills or protective factors, to research aimed at detecting the ‘state of resilience’, i.e. whether people can be considered resilient or not at a given time and on the basis of which characteristics. In this review, we therefore focused on the specific objects of study proposed by the authors: which aspects of resilience were the object of their work? To what extent did their findings contribute to the understanding of resilience and especially resilience as applied to psychosocial interventions targeting migrant populations?

### Procedure

In the bibliographic survey on the relationship between resilience, psychosocial intervention and migration, only empirical studies published in peer-reviewed journals were included. Books, book chapters, conference proceedings, reviews, editorials, reports, dissertations and other similar publications were excluded.

Only articles written in English and in French were included. Year constraints were used; the publication range was from 1993 to 2019. This choice was made because 1993 represents the year in which the first theoretical publications on resilience began to provide a conceptualisation of the construct [16]. The search terms were chosen according to the search requirements, and they were; resilience AND migration AND intervention; resilience AND adult AND asylum seekers OR refugees; resilience AND immigration AND adult; resilience AND immigrants AND intervention; resilience AND emigrants. The search was conducted in four databases: PsychInfo, Web of Science, Scopus and Pubmed.

In none of the articles obtained in the database research was found the term emigrant. Therefore, it would not seem to correspond to a category of research recognised in the literature.

Regardless of specific characteristics, the studies included concerned only adult participants. Studies with samples of minors were excluded from the research, being a population that requires specific attention [11, 17]. In fact, the international community, through the International Convention on the Rights of the Child (1989), has established specific rights and needs to children precisely because of the stage of development in which they find themselves. Consistently, the reception of these minors requires highly specific intervention strategies, actions and projects and, in all likelihood, an equally specific study. Researches conducted using a qualitative, quantitative and mixed method were included.

This systematic review was conducted in accordance with PRISMA guidelines (http://prismastatement.org/).

Proceeding with these methods, a total of 4.242 citations was obtained. After a first reading of the title, abstract and keywords, 3.944 articles were excluded. Despite advanced search filters, most of the search results did not meet the selection criteria described before. In most of the articles excluded, the relation between resilience and migration was not studied. Furthermore, many publications about resilience correlated to medicine, veterinary medicine, anthropology or sociology were excluded.

In many articles excluded, the function of the mother–child dyad in the resilience process was investigated. 148 articles were excluded because of duplicates.

**Fig. 1:**
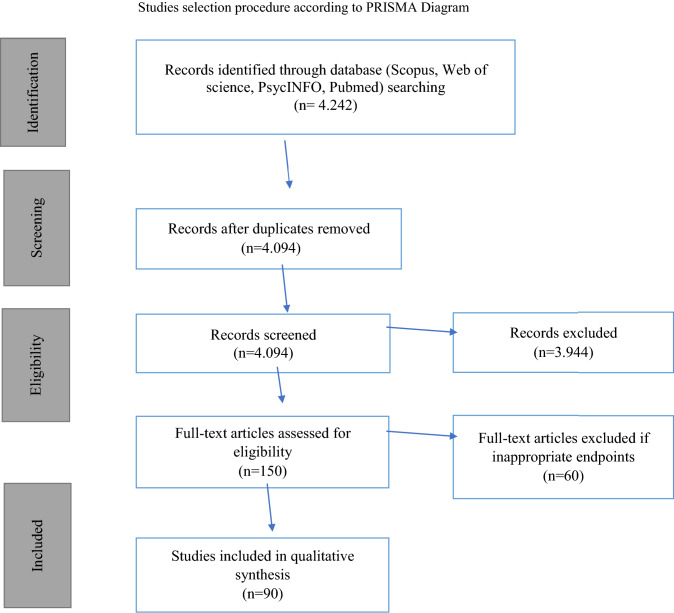
Studies selection procedure according to PRISMA Diagram

150 full-text articles were assessed for eligibility and 60 of them were excluded because they did not fulfil the eligibility criteria. As a result of these exclusions the publication time range was also reduced. 90 studies were included in this systematic review: 89 articles fall within a publication range from 2000 to 2019 and only one article dated 1993 (Fig. 1).

Three independent reviewers revised all abstracts and full-text analysed, and disagreements were resolved via group discussion. All authors were involved in all parts of the research.

The selected articles were uploaded on Nvivo 12 Plus and after an accurate reading, text extracts were aggregated into 4 main analysis categories, called main nodes in the software, and corresponding to our 4 analysis questions: participants, definition of resilience, object of study and intervention. Subsequently, a content analysis was made at each main node and organised into subordinate nodes. The Nvivo 12 software allows easy and systematic management of a large amount of textual material. Giving the possibility to work simultaneously on the main sources (the articles) and on the text sections, never losing the link between them (from source to extract and vice versa), Using Nvivo 12 reduces the risk of information loss.

## Results

### Participants

Participants were organised following gender and migrant’s status. The latter included economic migrants, asylum seekers and refugees.

#### Gender

In 47 articles, both men and women were included, among which the prevalence of participants was male. However, in these studies gender was a purely descriptive dimension. With only two exceptions [18, 19], in none of them was gender operationalised and the results obtained were not explained on the basis of this dimension.

In 29 studies, only women were involved. Consequences due to the violence experienced, sexual violence and exploitation, experience of pregnancy and childbirth in a foreign country, pregnancy followed by sexual violence, international marriages and the difficulty for women to access mental health services are the most studied topics in these works [20–29].

In other four studies [20, 30–32], only men were involved. Three of them did not explain why they chose male participants. One author addressed the issue of intersectionality, included gay Latino men in order to highlight the need to consider the risks of multiple discrimination that these people may also experience from their communities of reference, even in the context of migration.

In the remaining 10 articles, gender was not described.

#### Economic Migrants

Many studies implicitly referred to those who are legally identified as economic migrants. In fact, participants were described as migrants on the basis of their country of origin. They mainly belong to the most representative community of migrants in the context considered [19, 23, 25, 27, 28, 30, 33–58].

In other studies, the authors did not provide any further specification of the specific country and described the origin of the participants by referring to vast geopolitical areas such as "Latinos/as immigrants", "south Asian immigrant", "African immigrants", "southeast immigrants", "northeast immigrants", "former USSR immigrants", "Asian Indian immigrants", "Asian and African American immigrants" [32, 59–71].

In few studies, broader descriptions to vast geopolitical areas were made and, then, the countries of origin were deeper highlighted [60, 72–75].

Students [67] as well as the so-called "internal labour migrants" [47] are considered the same as migrants looking for new opportunities even if they move within their own country.

Two studies [26, 76] involved migrants, social workers and care professionals. In studying main factors of resilience in migrants, the authors included the point of view of social workers. Their experience could reveal vulnerable or strength points in institutional paths.

#### Asylum Seekers and Refugees

In a second group of studies, the description of the origin country was usually related to the legal status of participants [18, 20–22, 24, 29, 77–96]. The authors highlighted the difference of existential experience related to uncertain asylum seekers status and final legal refugee status. So, they described their post-migration status.

The asylum seekers’ conditions, characterised by uncertainty, difficulty to obtain a work permit and citizenship document in the host country, lack or impossibility of employment, could determine high levels of psychological disease in this population target [20, 22, 97]. The asylum seekers were described at a higher risk of developing psychological suffering than the other category of migrants [97].

Concerning refugees, even if they have a final legal status, authors emphasised the suffering due to the impossibility of return’s perspective, the lack of social support, the feelings of isolation and alienation.

Despite these deep different existential conditions, in a smaller group of studies, authors did not take into account the difference between refugees and/or asylum seekers and migrants [98–101].

### The Definition of Resilience

The analysis of the 90 publications allowed to distinguish three groups of articles: the first group included the articles with a not explicit definition of resilience, in the second group resilience was defined as coping or personality trait and in the last articles’s group authors defined resilience as a process (Table 1).

**Table 1:** Overview of included studies (N = 9063)

Author	Country	Type of article	Construct of resilience	Methodology	Participants	Genre
Abraham & Holtzkamp (2007)	South Africa	Research article	Process	Quantitative	Immigrati	M(12); F(56)
Abraham, Lien & Hanssen (2018)	Norway	Research article	Coping/personality trait	Qualitative	Asylum seekers	F(18)
Agani, Landau & Agani (2012)	Albania	Research article	Process	Intervention: LINC (Linking Human Systems Community Resilience model)	Refugees	M & F
Ahmad, Rai, Petrovic, Erickson & Stewart (2013)	Canada	Research article	Process	Qualitative	Immigrants	F(11)
Ajdukovic et al. (2013)	Balkan country	Research article	Coping/personality trait	Qualitative	Refugees	M(23); F(20)
Akinsulure-Smith (2017)	U.S.A	Research article	N.D	Qualitative	Immigrants	M(20); F(18)
Alajarian (2019)	London	Research article	N.D	Qualitative	Refugees	M(1); F(1)
Anderson, & Doyal (2004)	England	Research article	N.D	Mixed	Immigrants	F(62)
Araya, Chotai, Komproe & de Jong (2011)	Ethiopia	Research article	N.D	Qualitative	Asylum seekers	M(451); F(74)
Arenliu, Weine, Bertelsen, Saad & Abdulaziz (2019)	Turkey	Research article	Process	Qualitative	Refugees	M(n.d.); F(n.d.)
Arnetz, Rofa, Arnetz, Ventimiglia & Jamil (2013)	Australia	Research article	Coping/personality trait	Quantitative	Refugees Immigrants	M(52); F(76)
Aroian & Norris (2000)	U.S.A	Research article	Coping/personality trait	Quantitative	Immigrants	M(47); F(53)
Atallah (2017)	West Bank	Research article	Process	Qualitative	Refugees	M(n.d.); F(n.d.)
Aubé, Pisano & Merry (2019)	Canada	Research article	Process	Qualitative	Immigrant,Asylum seekers, Refugees	F(9)
Bajwa, Abai, Couto, Kidd, Dibavar & McKenzie (2018)	Canada	Intervention article	Process	Intervention: PSYCAP	Refugees, Care professional	M(19); F(24); Prof.(10)
Beiser, Simich, Pandalangat, Nowakowski & Tian (2011)	Canada	Research article	N.D	Quantitative	Refugees	M(54%); F(46%)
Beitin & Allen (2005)	U.S.A	Research article	Process	Qualitative	Immigrants	M(9); F(9)
Bender & Castro (2000)	U.S.A	Research article	Coping/personality trait	Qualitative	Immigrants	F(6)
Bogenschutz (2014)	U.S.A	Research article	N.D	Qualitative	Immigrants	M(5); F(4)
Brailovskaia, Schönfeld, Kochetkov & Margaf (2019)	U.S.A. e Russia	Research article	Process	Quantitative	Immigrants	M(304)
Bromand et al. (2012)	Germany	Research article	Coping/personality trait	Quantitative	Immigrants	F(105)
Christofer (2000)	U.S.A	Research article	Process	Quantitative	Immigrants	M(27; F(73)
Davis (2000)	U.S.A	Research article	N.D	Qualitative	Refugees	F(19)
Denov, Fenning, Rabiau & Shevell (2019)	Canada	Research article	Process	Qualitative	Refugees	M(1)
Ellis, et al. (2015)	U.S.A	Research article	Process	Mixed	Refugees	M(233); F(141)
Febres-Cordero, Brouwer, Rocha-Jimenez, Fernandez-Casanueva, Morales-Miranda & Goldenberg (2018)	Mexico	Research article	N.D	Qualitative	Immigrants	F(31)
Fino, Mema & Russo (2020)	Albania	Research article	Coping/personality trait	Quantitative	Refugees; Asylum seekers	M(76); F(7)
Gagnon & Stewart (2014)	Canada	Research article	Process	Qualitative	Immigrants, Refugees; Asylum seekers	F(10)
Goodman, Vesely, Letiecq & Cleaveland (2017)	U.S.A	Research article	Process	Qualitative	Immigrants; Refugees	F(19)
Gray, Mendelsohn & Omoto (2015)	U.S.A	Research article	N.D	Qualitative	Immigrants	M(13)
Guruge, Maheu, Zanchetta, Fernandez & Baku (2011)	Canada	Research article	N.D	Qualitative	Immigrants	F(12)
Hernandez & Moreno (2014)	Spain	Research article	Process	Qualitative	Immigrants, Care professionals	M(n.d.); F(n.d.)
Hoffman, Stich, Musani & Robertson (2019)	Burma	Research article	N.D	Qualitative	Refugees	M(9); F(26)
Hooberman, Rosenfeld, Rasmussen & Keller (2010)	U.S.A	Research article	Coping/personality trait	Quantitative	Refugees	M(44); F(31)
Hosseini, et al. (2017)	Australia	Research article	Process	Quantitative	Immigrants; Refugees	M(99); F(83)
Hwahng, Allen, Zadoretzsky, Barber, McKnight & Des Jarlais (2019)	New York	Research article	Coping/personality trait	Qualitative	Immigrants	F(13)
Jackson, et al. (2004)	U.S.A	Research article	N.D	Quantitative	Immigrants	n.d
Jowell (2018)	Tanzania	Research article	Process	Qualitative	Immigrants	F(31)
Khawaja, Ramirez & Prasad-Ildes (2013)	Australia	Intervention article	Coping/personality trait	Intervention: BRITA	Immigrants: Refugees	M(12); F(29)
Klokgieters, van Tilburg, Deeg & Huisman (2018)	Netherlands	Research article	Process	Qualitative	Immigrants	F(680)
Kuo et al. (2019)	Taiwan	Research article	Coping/personality trait	Quantitative	Immigrants	F(118)
Kwong, Du & Xu (2015)	U.S.A	Research article	Coping/personality trait	Qualitative	Immigrants	M(7); F(10)
Lee, Brown, Mitchell & Schiraldi (2008)	U.S.A	Research article	Coping/personality trait	Quantitative	Immigrants	M(170); F(200)
LeMaster, et al. (2018)	U.S.A	Research article	Coping/personality trait	Quantitative	Refugees	n.d
Lemus-Way & Johansson (2019)	U.S.A	Research article	Process	Qualitative	Immigrants	F(12)
Li, Xu & Chi (2018)	Los Angeles	Research article	Coping/personality trait	Qualitative	Immigrants	M(11); F(13)
Liang, Teng & Xu (2019)	China	Research article	Coping/personality trait	Quantitative	Immigrants	M(622); F(90)
Lim & Han (2016)	South Korea	Research article	Coping/personality trait	Quantitative	Refugees	M(107); F(338)
Lusk, Terrazas, Caro, Chaparro & Antùnez (2019)	Central America	Research article	Process	Qualitative	Immigrants	n.d
Margherita & Tessitore (2019)	Italy	Research article	Process	Quantitative	Asylum seekers	M(20)
Marsiglia, Kulis, Perez & Bermudez-Parsai (2011)	U.S.A	Research article	Coping/personality trait	Quantitative	Immigrants	F(136)
Melamed, Chernet, Labhardt, Probst-Hensch & Pfeiffer (2019)	Switzerland	Research article	Process	Qualitative	Asylum seekers	F(12)
Mendelsohn, et al. (2014)	Kenya-Malesia	Research article	Coping/personality trait	Qualitative	Refugees	M(12); F(14)
Miller & Chandler (2002)	U.S.A	Research article	Coping/personality trait	Quantitative	Immigrants	F(200)
Moore (2018)	U.K	Research article	Coping/personality trait	Quantitative	Immigrants	M(32); F(19)
Nam, Kim, DeVylder & Song (2016)	South Korea	Research article	Coping/personality trait	Quantitative	Refugees	n.d
Nesteruk (2017)	U.S.A	Research article	N.D	Qualitative	Immigrants	M(13); F(46)
Nguyen, Stanley, Stanley & Wang (2015)	China +	Research article	Process	Mixed	Immigrants	M(9); F(11)
Obrist & Buchi (2008)	Switzerland	Research article	Process	Qualitative	Immigrants	M(11); F(9)
Orton, Griffiths, Green & Waterman (2012)	U.K	Research article	Process	Qualitative	Asylum seekers	n.d
Pan (2015)	Australia	Research article	Coping/personality trait	Quantitative	Immigrants	M(75); F(152)
Peddle (2007)	U.S.A	Research article	N.D	Quantitative	Refugees	M(34); F(49)
Ponizovsky-Bergelson, Kurman & Roer-Strierv (2015)	U.S.A	Research article	Process	Quantitative	Immigrants	M(156); F(64)
Poudel-Tandukar, Chandler, Jacelon, Gautam, Bertone-Johnson & Hollon (2019)	U.S.A	Research article	Coping/personality trait	Quantitative	Refugees	M(113); F(112)
Ritsner,et al. (1993)	Israel	Research article	N.D	Quantitative	Immigrants	M(152); F(233)
Roberto & Moleiro (2015)	Portugal	Research article	Process	Qualitative	Immigrants	M(9); F(26)
Rocha-Jimenez Brouwer, Silverman, Morales-Miranda & Goldenberg (2016)	Guatemala	Research article	N.D	Qualitative	Immigrants	F(52)
Salgado et al. (2014)	U.S.A	Research article	N.D	Quantitative	Immigrants	M & F = 650
Schweitzer, Greenslade & Kagee (2007)	Australia	Research article	Coping/personality trait	Qualitative	Refugees	M(10); F(3)
Serafica, Lekhak & Bhatta (2019)	U.S.A	Research article	N.D	Quantitative	Immigrants	n.d
Silva, Paris & Anez (2017)	U.S.A	Intervention article	Coping/personality trait	Intervention: CAMINO	Immigrants	n.d
Singh, Hays, Chung & Watson (2010)	U.S.A	Research article	Coping/personality trait	Qualitative	Immigrants	F(13)
Siriwardhana, Abas, Siribaddana, Sumathipala & Stewart (2014)	Sri Lanka	Research article	Process	Quantitative	Immigrants	M(166); F(284)
Stempel, Sami, Koga, Alemi, Smith & Shirazi (2017)	U.S.A	Research article	N.D	Quantitative	Refugees	M & F
Tippens (2017)	Kenya	Research article	Coping/personality trait	Qualitative	Refugees	M(27); F(28)
Torres, et al. (2016)	U.S.A	Research article	N.D	Quantitative	Immigrants	n.d
Tummala-Narra, Sathasivam-Rueckert & Sundaram (2012)	U.S.A	Research article	N.D	Qualitative	Immigrants	M(8); F(10)
Vahabi, Wong & Lofters (2017)	Canada	Research article	N.D	Mixed	Immigrants	F(30)
Van der Ham, Ujano-Batangan, Ignacio & Wolffers (2014)	Filippine	Research article	Process	Mixed	Immigrants	F(500)
Wong & Song (2008)	China	Research article	Process	Quantitative	Immigrants	M(236); F(238)
Wright et al. (2017)	U.S.A	Research article	Coping/personality trait	Mixed	Refugees	M(158); F(133)
Wu,Y. & Wu, H. (2015)	Taiwan	Research article	N.D	Qualitative	Immigrants	F(11)
Xin, Aronson, Lovelace, Strack & Villalba (2013)	Vietnam	Research article	Process	Qualitative	Refugees	M(15); F(20)
Yakushko & Morgan-Consoli (2014)	U.S.A	Research article	N.D	Qualitative	Immigrants	F(8)
Yamanis et al. (2018)	U.S.A	Research article	N.D	Quantitative	Immigrants	n.d.
Yu, Lam, Liu & Stewart (2015)	China	Research article	Coping/personality trait	Quantitative	Immigrants	F(220)
Yu, Stewart, Chui, Ho, Li & Lam (2014)	China	Research article	Coping/personality trait	Quantitative	Immigrants	M(45); F(175)
Yu, Stewart, Liu & Lam (2014)	China	Research article	Coping/personality trait	Quantitative	Immigrants	M(384); F(921)
Yung, et al. (2015)	Taiwan	Intervention article	Coping/personality trait	Intervention: action research	Immigrants	F(68)
Zaheer, Eynan, Lam, Grundland & Links (2018)	Canada	Research article	N.D	Qualitative	Immigrants	F(10)

In the first typology of articles [21, 27, 28, 32, 34, 37, 38, 44, 46, 51, 56–58, 60, 63, 66, 69–71, 78, 79, 83, 86, 87, 94, 102] authors did not give an explicit definition of resilience. They indicated resilience either in their aims or results, as a factor to study well-being, mental health or illness in the migration process without referring to a precise theoretical model. Despite the lack of definition of the construct, authors [38, 69, 71, 78] identified internal and external factors or resources that could favour resilience, such as locus of control, social support and workplace or household satisfaction. With reference to the specific topic of migration, authors identified several sources of resilience, depending on different motivations of departure, volunteer or forced, attitude of migrants on the migration process and level of identification with the host culture and its values like resilience’s personal and family resources and the possibility to send money to family member [38, 69].

In the second group of articles a growing consensus emerged that considers resilience and coping or personality trait as closely related [22–25, 29, 33, 35, 39, 42, 49, 50, 55, 61, 62, 65, 74, 75, 81, 82, 84, 85, 90, 91, 93, 96, 98, 101, 103–105]. Resilience is then defined as patterns of responses that aid to explore and negotiate social, political and environmental resources [85] and through some coping abilities, such as strength, courage and perseverance that characterise an individual during change [74].

Furthermore, some authors [29, 33, 65, 96, 98] defined resilience as a personality trait that helps the individuals to protect themselves from psychological disorders resulting from exposure to violent incidents. In addition, resilience minimises the negative effects of stress and promotes adaptation. Therefore, resilience was conceived as a stable element of personality like locus of control, introversion, extraversion and self-efficacy that could influence immigrant adaptation (103).

While for some authors [49, 75, 81, 91, 105] resilience was defined as a functional ability employed in migrant's past traumatic experiences; for others, resilience referred to the ability to cope with future adversities [101]. Therefore, in some studies [91, 93, 101] resilience was conceptualized as a tool to support a broader process of acculturation or adaptation.

The last group conceptualised resilience as a process. In these studies, authors utilised theoretical models that define resilience as a dynamic process in which psychological, social and environmental factors give a not pre-established trajectory to individuals or families during time. This favours the opportunity to regain, maintain or develop their well-being despite adversities [18–20, 26, 30, 31, 36, 40, 41, 41, 43, 47, 48, 52–54, 59, 64, 67, 68, 72, 73, 76, 80, 88, 89, 92, 95, 97, 99, 100, 106, 107]. The literature has highlighted a wide range of factors that protect and promote the resilience process. According to the authors, individuals were not only able to cope and adapt to adverse conditions (reactive capacity), but also to seek and create options (proactive capacity). In this way, they develop greater competence (positive outcomes) in dealing with a threat or risk. The individuals were able to make decisions by using the available resources to overcome the adverse or traumatic experience [20, 40, 41, 43, 47, 64, 73].

Other authors [80] highlighted among the social factors, family cultural values, the possibility of mutual psychological support and strong social ties. Also, according to authors (72, 19] resilience was a process influenced by the good functioning of the family, the members' ability to make sense of the crisis from past and present experiences, to stay connected, to cope with the crisis, and to move forward in their lives. In other cases, therefore, family resilience was explored by particular characteristics of the family like their belief systems, organizational patterns, and communication processes [88].

### Methodological Approaches

About the methodological approaches, a first distinction between research articles (85 articles) and intervention strategies (5 articles) was made.

Subsequently, a further distinction between qualitative vs quantitative approach in the 85 research articles was presented.

Articles with a qualitative methodological approach were 40: individual interviews [18, 20, 24, 27, 30, 33, 35, 40, 48, 52, 54, 57, 58, 61, 75, 80, 86, 88, 89, 95, 101], focus groups [25, 41, 62, 71, 73] and participating observations [26, 56, 96]; furthermore, 5 studies [21, 22, 29, 62, 99] used both in-depth interviews and focus groups and 3 studies [56, 76, 92, 103] both semi-structured interviews and participating observation. Finally, in 2 articles [21, 86] a case study method was utilised.

A primary aim of the authors that have chosen a qualitative methodological approach was to highlight the narration of individual experiences. Indeed, some authors included the entire life history and migration experience in their analysis. In this way, three main moments (before migration—during migration—after arrival in the host country) were identified [20, 30, 35, 41, 57, 58, 61, 62, 86, 99, 104]. Some others [22, 76] focused on the whole migration experience. In their study, authors [73] preferred to conduct the analysis starting from the two main thematic areas: the arrival and time spent in the host country and the resources and strategies used to cope with these difficulties. Other authors preferred to explore specific themes with more targeted stimulus questions to identify participants’ personal, family, social and contextual resources and strategies fostering resilience [18, 21, 24, 25, 29, 33, 40, 48, 75, 96].

About the language of interview, the participants' native language was preferred by the authors. Subsequently, the materials were translated into English [75, 84, 104].

About data analysis procedure, in some cases the authors followed specific models of qualitative analysis as phenomenological approaches [18, 57, 80, 104]. However, the preferred analysis approach was a content analysis.

Articles with a quantitative method were 35. In these articles, many different tests about resilience and other psychological and clinical dimensions were used. Furthermore, some studies investigated resilience starting from the family resilience framework.

The main standardized resilience tests utilised were the Resilience Scale (RS), the Connor-Davidson Resilience Scale (K-CD-RISC) and Brief Resilience Scale (BRS) [39, 49, 72, 84, 85, 98]. In a study [28] was described the Multidimensional Trauma Recovery and Resiliency Scale (MTRR) that measures trauma and resilience in refugee populations. Additionally, authors reported a positive and therapeutic function of the standardized interview as reported by participants themselves. In another study [29] that aimed to investigate the life condition of Southeast Asian immigrant women who divorced in Taiwan, authors wanted to develop and test a Chinese version of Resilience Scale. According to the authors, there are some differences between divorced Southeast Asian immigrant women and divorced women in other populations. These differences were explained because of their different cultural and gender values. The final 16-item RS-C resulted in a three-factor model (personal competence, family identity and social connections). However, authors suggested additional research on the RS-C to further establish its reliability and validity.

The following questionnaires were used to investigate other psychosocial dimensions studied in the migration process: Migration and Settlement Questionnaire, Questionnaire Social Support, Social Support Index, Relation and Friend Support Index, Subjective Happiness Arab Acculturation Scale, the Demands of Immigration (DI) scale, the Migration Quality of Life Scale and Social Provisions Scale. In this way, authors aimed to measure the stress of acculturation, the perception of social exclusion and discrimination, the process of personal and family resilience [82, 85, 91, 93].

In addition, some studies often integrated psychosocial tests with clinical tests to measure the specific mental health status. The main tests were: Depression Anxiety Stress Scale, Harvard Trauma Questionnaire, 90-item Symptom Checklist (SCL-90-R), General health questionnaire (GHQ-12). For example, authors [106] used an online questionnaire, proposed both in English and Farsi, which included questions on migration experience, levels of depression and resilience.

In the studies that investigated resilience in the family context utilised specific measurement: the Family Problem Solving Communication Index (T-PSC), Family Hardiness Index, F-COPES, Family Time and Routine Index (FTRI), Family Attachment and ChangeaBility Index 8.

Finally, mixed-methodology studies (10 articles) aimed to integrate personal and narrative results [51] and statistical data obtained through standardized tests [31, 36, 64, 68, 97, 100]. In their study, authors [43] used data collected through questionnaires to build workshops or focus groups for the second qualitative phase of their study. In another study [45] the interviews were used to create ad hoc scales in line with the participants' reports.

### Intervention Procedures

As anticipated, only 5 articles described the intervention strategies aimed to measure, enhance or foster the resilience process in the migration context [18, 74, 101, 105]. Two of them [74, 105] involved only groups of economic migrants. In the other two articles [18, 74] only refugees were included. The last work [101] involved both economic migrants and refugees.

The first publication [18] described a model of intervention called LINC (Linking Human Systems Community Resilience) and applied in post-war Kosovo following emergencies of worrying levels of circulation and abuse of substances, especially among the younger generation after the population’s return in the origin country. These generations seemed to be the most vulnerable target, because they were influenced by a highly tragic collective history inscribed in the past of the community itself. According to authors, this type of intervention was used in different communities around the world where drastic and sudden political changes or environmental disasters occurred. In these cases, there was an increase in the needs of the population, especially in terms of mental health. According to authors, LINC Community Resilience extends the concept of resilience to the level of community encouraging people to view themselves as competent in the face of overwhelming circumstances. Authors strengthened protective resources already present in the community to protect it by risk factors such as the feeling of shame and the sense of threat to the family identity. According to authors, family cohesion, the main historical and cultural values of the community, the positive function of these values’ transgenerational narrative and the attribution of new meanings to the tragic past’s events were considered protective factors. Authors highlighted that the imposition of interventions culturally distant from the context of application was avoided. As a result of LINC intervention, the creation of a Centre for treatment, education and research on addiction have been seen. This centre worked on a program that provides specific training for professionals to strengthen the community's resources: local multicultural skills, awareness of one's own values, strengths, prejudices and use of one's own skills (concreteness, authenticity, and self-disclosure) to build a relationship of trust. In this way, the client and family could share their stories to build sustainable recovery.

The authors of the second publication [101] drew on previous studies indicating that people with language barriers have the greatest difficulty integrating into Australian society. At the same time, they noted the lack of specific language learning programmes aimed at the adult migrant population. They therefore set out to strengthen the resilience process of adult migrants in Australia by implementing a culturally sensitive language learning programme. The participants of their study were migrants and refugees, both men and women, from 30 different countries all over the world, except North America. BRiTA is a set of modules that aims to encourage the emergence and the sharing in groups of the protective factors and resources already present in each person. Following the application of BRiTA Future, the authors [56] observed a reduction of the risk of marginalization through this sharing. The authors highlighted the need for more rigorous measurements.

In the third research paper [74] was described the CAMINO intervention tool developed for Latinos community. According to authors, migrants from South and Central America (Mexico, Cuba, Dominical Republican, Puerto Rico) to the United States represented the majority of the migrant population. Their great need to access the health care system poses a critical issue because of the complexity of their migratory experience. Despite efforts to recognise their cultural strengths and vulnerabilities, authors highlighted the need to develop culturally-sensitive psychosocial and clinical interventions. According to authors, CAMINO was a culturally-sensitive tool. It facilitated the selection and evaluation of key elements of each participant's pre and post migration experiences: the impact of community and family support, acculturation stress level, history of migration, language preference, idioms of distress and resilience, origins. In this program, resilience was a subcategory that defined the participants' response in extreme situations of difficulty. This response will depend on the participants' perception of their own strength, their sense of mastery and their expectations for a definitive solution. CAMINO consisted of six macro-areas investigated through a series of questions, recommended by the authors. These areas were not exclusive and do not follow a pre-established order. This article described the intervention procedure, without reporting the results of its implementation.

Instead, the fourth intervention [105] aimed to improve life satisfaction and guarantee good higher educational goals for refugee survivors who participated in educational programs in Canada. According to authors, the traumatic experience and the language difficulties hinder social inclusion. To promote it and favour life satisfaction is important to foster psychological capital. According to the authors, resilience is the fourth dimension of a greater psychological capital’s concept, together with hope, efficacy and optimism. The intervention was divided in three phases: a preliminary group discussion to explore needs, barriers, expectations, and experiences that refugees who are trauma-survivors faced in pursuing higher education in Canada. In the second phase, these shared needs determined the content of the educational program’ sessions. It consisted of a 14-weeks course organized into weekly four-hour workshops that covered pertinent topics emerged by group discussions. In the third phase, participants have started the educational program’s structured course. Furthemore, authors have administered psychosocial tests to measure their self-esteem, psychological capital, life satisfaction, and level of participation in their respective communities at three different moments (entry point -week 1, mid-point -week 8, and exit point -week 14). The goal of the last phase was to give resources and educational support to respond to refugees' own needs to help them identify and pursue their social, educational, and vocational trajectories in the host country. Authors reported as a result that the participants’ psychological capital, specifically the dimensions of resilience and optimism, was positively correlated with their life satisfaction. In this study, authors did not describe the content of the educational program, but it only reported its results.

Finally, the last intervention [72] was an action research aiming to promote health empowerment for women who migrated in Taiwan from Vietnam, Indonesia, Philippines, Thailand and Cambodia and engaged in transnational marriages. The growing number of transnational marriages had a great impact on social and public health. According to authors, these women suffered intersectional discrimination for both marriage and migration experiences in the host country. The authors wanted to promote a deep sharing of the meanings among participants and their resignification of adverse and/or traumatic events to make sustainable and beneficial changes in their health and well-being. Authors reported four areas of intervention to gain the aim: increasing health literacy, facilitating the ability to build social networks, increasing the sense of self-esteem and building psychological resilience. Therefore, they described the need to build psychological resilience to transform life distress of women and their children into a more positive future outlook. The intervention promoted the participants' ability to identify problems and work together to find a solution.

## Conclusions

This systematic review aimed to contribute to a better and more focused understanding of the link between the concept of resilience and psychosocial interventions in the migrant population.

The emergency of the migration issue, on the one hand, impacts a large proportion of the world’s population in both the roles of host and guest, and, on the other hand, produces a vast amount of work in academia. This led us to delve into the literature in order to outline the information that would be useful for the definition of practical interventions for the benefit of individuals and communities.

Looking at the vastness of the existing literature, there was a need to somehow systematise what had been studied in this specific field.

What have we learned from these studies?

The participants involved in these studies are mainly defined according to formal and legal criteria, and they can be easily distinguished into economic migrants, asylum seekers and refugees.

This distinction positions migrants in relation to the host country, but offers very little information in relation to their migration experience [108].

This categorisation criterion [109], leads to a homogenisation of the target group that risks obscuring the recognition of the needs of the individuals.

If all migrants are united by the fact that they have experienced migration, each of them has had a personal experience of it, rooted in his/her personal life.

Similarly, it appears that in the description of participants the different geopolitical origin and cultural specificities have not been adequately taken into account. Yet it is precisely in these personal experiences, which also take on significance depending on cultural background, that possible resilience factors can be identified. What happened before I left? Who do I leave behind? Who will be able to greet me on arrival? What are my chances of coming back? What did I risk to leave?

These are all existential questions that should allow psychologists to better define migrants' potentials risks and resources, regardless of the legal category to which they have been attributed.

The studies consulted in this review, whether conducted with qualitative or quantitative methodologies, tend to measure, at a given time, stable dimensions as strength, perseverance, courage, self-esteem, self-discipline, self-control, self-efficacy, capacity for action, ability to solve problems or manage stress. When considered, the more social dimensions that might influence resilience or its process are social support, workplace or household satisfaction and community ties. It seems also that, on a family level belief system, remittances to family in the origin country, good functioning, organizational pattern and communication processes of family are all favorable conditions for resilience. On the contrary, dramatic departures, bereavements, difficult identification with the host culture, sense of alienation, difficulty in accessing employment, health and social services are risk factors that may undermine resilience or its outcomes.

On the basis of this information, we should deduce that if a person feels strong, capable, competent, able to cope with problems and able to solve them, if he/she has not had to face too traumatic or adverse experiences, if he/she has a good family and social support network, then, in all likelihood, we can consider him/her a resilient person.

This information, which inevitably sounds a bit tautological, can be applied to any existential context and informs us very little about the migrant's condition and how it would be useful to intervene in order to promote the process of resilience and thus his/her well-being.

As a matter of fact, resilience’s construct is difficult to operationalise because of its polysemic definitions. It should also be noted that a large number of publications refer to the construct of resilience without making the theoretical framework explicit. This difficulty determined the main obstacle to plan efficiency interventions [13]. In migration contexts, there are few works that described resilience interventions. They mostly were group-oriented, enhancing personal and social factors already present in the participants' life stories and co-constructing the procedure’s priorities. The interventions aimed to foster migrants’ resources or factors that involve psychosocial dimensions of the migration process, such as migrant’s social ties, agency, proactive capacity and their knowledge of language, cultural values or health and social system of host countries [110].

The results of resilience studies did not translate into a clear prevention and treatment programme [111] and did not adequately address the need to re-elaborate the trauma that could characterise the migration experience in most cases. On one hand, the not well-described procedures hinder other professionals from adopting them. Specifically, psychosocial dimensions of the migration process—migrant’s awareness of culture, cultural aspects (such as norms, customs, language, lifestyle, social contest) and their capacity to distinguish between culture and pathology—in the interventions’ planification have not been described [14]. Often, the intervention’s efficiency and implication for the population involved have not been reported.

On the other hand, little attention was paid to the need to overcome trauma to foster resilience. As argued in the literature [111, 112], through clinical and psychosocial interventions, it was important to foster emotional support and genuine encounters with peers or care professionals in order to aid people to deal with pain, loss and injury related-trauma favouring a positive future outlook. The resilience interventions should take into account a relational perspective to try to rebuild migrants’ new life [113, 114] and to obtain a new balanced-psychosocial health. Therefore, a full view of the resilience strategies’ impact to promote a general psychosocial health status and wellbeing was not inferred.

Furthermore, within the studies’ participants a lack of involvement of care professionals working with migrants has emerged. They are a key element in the relational care process with the hosted migrants. These professionals co-build a relationship within the health and support system to favour migrants’ resilience trajectory [112]. The involvement of professionals should be an integral part of the future research. We can therefore assume that the success of the intervention will also partly depend on the outcome of this specific helping relationship.

This systematic review has some limitations. The search terms selected and used in the review could limit the number of results, although we tried several different combinations to avoid it. The search term “immigrant” does not seem to be adequate to review’s aim because it does not correspond to a category of research. The research has been carried out choosing “intervention” as a keyword. Future research could expand the topic adding other literature relevant keywords.

Furthermore, only a qualitative analysis was conducted. To assess the efficacy of interventions to foster resilience, a more in-depth methodological analysis, such as a quantitative analysis of reported articles’ measures and results, could have been conducted.

This systematic review tried to offer an exhaustive picture of the literature on the promotion of resilience in the migratory context. It has highlighted a gap in the literature on this topic-related. A clear definition of resilience in the migration process could allow to recognise which protective factors and resources were relevant to plan other interventions. These should take into account the cultural appropriateness and the traumatic dimensions of the migratory experience. Finally, it could be relevant to incentivise the authors to complete exhaustive procedures for reliable interventions and measurement of their efficacy.
